# Organic polaritonic light-emitting diodes with high luminance and color purity toward laser displays

**DOI:** 10.1038/s41377-024-01531-0

**Published:** 2024-08-15

**Authors:** Jianbo De, Ruiyang Zhao, Fan Yin, Chunling Gu, Teng Long, Han Huang, Xue Cao, Cunbin An, Bo Liao, Hongbing Fu, Qing Liao

**Affiliations:** 1 Beijing Key Laboratory for Optical Materials and Photonic Devices, Department of Chemistry, Capital Normal University, 100048 Beijing, China; 2 Beijing Special Engineering Design and Research Institute, 100028 Beijing, China; 3 Institute of Process Engineering, Chinese Academy of Sciences, 100190 Beijing, China; 4 Institute of Molecule Plus, Tianjin University and Collaborative Innovation Center of Chemical Science and Engineering (Tianjin), 300072 Tianjin, China; 5 School of Materials Science and Engineering, Hunan University of Science and Technology, 411201 Xiangtan, Hunan China

**Keywords:** Organic LEDs, Microresonators

## Abstract

Achieving high-luminescence organic light-emitting devices (OLEDs) with narrowband emission and high color purity is important in various optoelectronic fields. Laser displays exhibit outstanding advantages in next-generation display technologies owing to their ultimate visual experience, but this remains a great challenge. Here, we develop a novel OLED based organic single crystals. By strongly coupling the organic exciton state to an optical microcavity, we obtain polariton electroluminescent (EL) emission from the polariton OLEDs (OPLEDs) with high luminance, narrow-band emission, high color purity, high polarization as well as excellent optically pumped polariton laser. Further, we evaluate the potential for electrically pumped polariton laser through theoretical analysis and provide possible solutions. This work provides a powerful strategy with a material–device combination that paves the way for electrically driven organic single-crystal-based polariton luminescent devices and possibly lasers.

## Introduction

In the past decades, organic light-emitting diodes (OLEDs) have made significant progress and shown great promise in display applications due to their ease of fabrication, lightweight flexibility, and high efficiency^1–4^. Nevertheless, most organic luminescent materials exhibit broad emission with full width at half-maximum (FWHM > 60 nm)^5,6^, due to the intrinsic exciton–vibration coupling and structural relaxation at the excited states^7–9^. This brings about poor color purity and inability to meet the International Telecommunication Union-Radiocommunication Recommendation BT.2020 color gamut standard in ultrahigh-definition (UHD) displays^10,11^. In recent years, the emerging hyperfluorescence and boron/nitrogen (B/N)-based polycyclic multiple resonance strategies have inspired OLEDs to narrow their FWHM to below 25 nm^10,12–14^. However, it remains a practical challenge in the synthesis of new molecules and in satisfying the color gamut standard of BT.2020.

The strategy of strongly coupling the organic exciton state to an optical microcavity provides an alternative solution, achieving the narrowband emission^15–17^. In such a strong coupling system, the strong hybridization between organic excitons and cavity photons leads to the formation of stable exciton–polariton (EP) quasiparticles at room temperature, benefiting from the large exciton binding energies and oscillator strengths of organic excitons^18,19^. Thanks to the advantageous features inherited from their constituents, EPs have emerged as an attractive platform for exploring the quantum phenomena as well as optoelectronic applications^20–22^. In particular, due to their bosonic characteristics, EPs can macroscopically condensate the polariton ground state and achieve coherent laser-like emission without population inversion at thresholds orders of magnitude below conventional photon laser^23–25^. Organic EP lasers, leveraging the high monochromaticity (FWHM of <1 nm), high luminosity, and extensive achievable color gamut, exhibit great potential for revolutionary display technologies^26,27^. However, the readily available organic EP-based electroluminescent (EL) devices, namely organic polariton light-emitting diode (OPLEDs), still suffer from low carrier mobilities, low external quantum efficiency (EQE), and poor stability at high current densities, resulting in the inability to reach the current densities required for EP condensation^22,28^. Hence, there is an urgent requirement for organic EP devices with high performances and low condensation threshold under electric pumping.

Organic single-crystals (OSCs) have shown attractive potential in EL devices due to their perfect crystallinity and excellent electrical and optical properties^23,25,29,30^. In this work, we demonstrate novel single-crystal OPLEDs with high luminance, narrow emission, and high polarization, as well as optically pumped polariton lasing at room temperature. The electrically pumped pure-red-color EP emission with the main band located at 627 nm and FWHM of 4.1 nm is clearly observed, exhibiting highly polarized degree of 170 and angle-independent high color purity with the Commission Internationale de l’Éclairage (CIE) chromaticity coordinates of (0.69, 0.31) due to excellent EP dispersion, which is close to the standard of BT.2020 of (0.708, 0.292). Thanks to the virtues of OSCs, including low defect density and balanced carrier transport, this OPLED displays a high luminance of over 780,000 cd m^−2^ and remarkable long-term stability. This device also demonstrates an excellent optically pumped polariton laser. We theoretically analyze and demonstrate that our OPLEDs are a promising way to the electrically pumped polariton laser. This work provides a powerful strategy with a material–device combination that paves the way for electrically driven OSC-based polariton luminescent devices and possibly lasers.

## Results

### Anisotropic strong coupling in an organic single-crystal microcavity

**Fig. 1:**
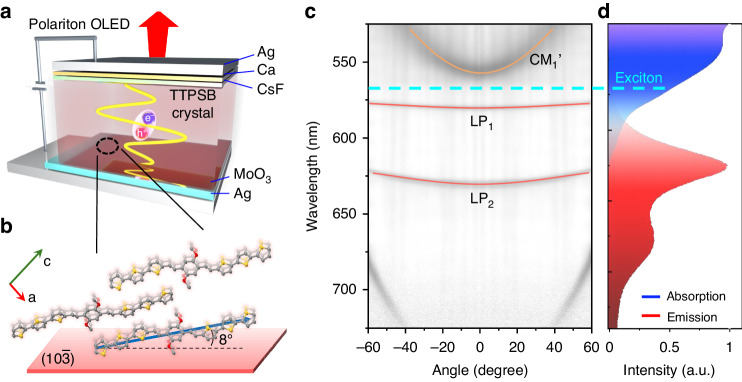
Schematics of OPLED structure and angle-resolved reflectivity. **a** Scheme of the TTPSB-microcrystal-based OPLED. **b** The molecular packing along [011] direction. The microcrystal is bounded by ($10\bar{3}$) and ($\bar{1}03$) crystal planes on the top and bottom surfaces. The transition dipole-moment of TTPSB (blue arrow) along the molecular long-axis is tilted at an angle of 8° with ($10\bar{3}$) crystal plane. **c** Angle-resolved reflectivity of the microcavity. LP_1_ and LP_2_ (red lines) are lower polariton branches caused by strong coupling between cavity modes and the 0-1 excitons at 570 nm (cyan dotted line). The CM_1_’ is in uncoupled cavity mode. **d** The absorption (blue line) and PL (red line) spectra of the TTPSB microcrystals

Figure 1a schematically shows the OPLED device architecture used in this work: silver (Ag, 150 nm)/molybdenum trioxide (MoO_3_, 5 nm)/1,4-dimethoxy-2,5-di(2,2’,5’,2”-terthiophenestyryl) benzene (TTPSB) OSC (~580 nm)/cesium fluoride (CsF, 2 nm)/calcium (Ca, 10 nm)/silver (Ag, 35 nm). Here, the upper and bottom silver films can not only serve as electrodes for charge injection but also as an optical microcavity based on the high reflectivity of silver film. The reflectivity of 150-nm Ag film is more than 99%, and that of 35-nm film reaches about 50%.

The synthesis and characterization of TTPSB microcrystals and TTPSB-based OPLEDs are detailed and described in the Supporting Information^31^. The as-assembled TTPSB microcrystals show uniform red photoluminescence (PL) and a smooth surface with a roughness of <1 nm (details in Fig. S1). As shown in Fig. 1b, TTSPB molecules are orderly stacked preferentially along the crystal [011] (defined as long axis) and [103] directions of the crystal. The transition dipole moment of TTPSB (blue arrow) is oriented parallel to the molecular long axis with an angle of 8° with ($10\bar{3}$) crystal plane (red plane). This near-in-plane orientation of dipole moments is conducive to the occurrence of anisotropic strong coupling and the enhancement of the outcoupling EL efficiency.

Figure 1c shows the unpolarized reflectivity as a function of the wavelength and angle of the sample, carried out by a homemade Fourier imaging spectroscopy setup covering the angle range of *θ* = ±60° at room temperature (details in Fig. S1). Two sets of modes with distinctive curvatures are observed, respectively, marked by an orange curve for one large curvature mode and red curves for two smaller curvature modes. Polarization-dependent angle-resolved reflectivity (Fig. S2) was performed by adding a linear polarizer in the detection optical path. These two sets of modes are attributed to two orthogonal polarizations, that is, the large-curvature mode is horizontal (H)-polarization (parallel to the long axis of the microcrystal), while the two small-curvature modes are vertical (V)-polarization (perpendicular to the long axis). The V-polarized modes can be well-fitted using a coupled harmonic oscillator Hamiltonian model (details in Supporting Information)^32^. The corresponding fit parameters are shown in Table S2. This result indicates that they originate from lower polariton dispersions (LP_1_ and LP_2_ in Fig. 1c), deriving from the strong coupling between two V-polarized cavity modes and organic excitons at the first excited singlet state (S_1_ at 573 nm), as shown in the absorption spectrum (blue line in Fig. 1d). Notably, the broad and strong absorption of TTPSB microcrystals causes the upper polariton branches to be invisible due to the nature of H-aggregation (the corresponding structural and spectroscopic analysis in Figs. S3 and S4)^31^.

### EP lasing through vibration-assisted relaxation

**Fig. 2:**
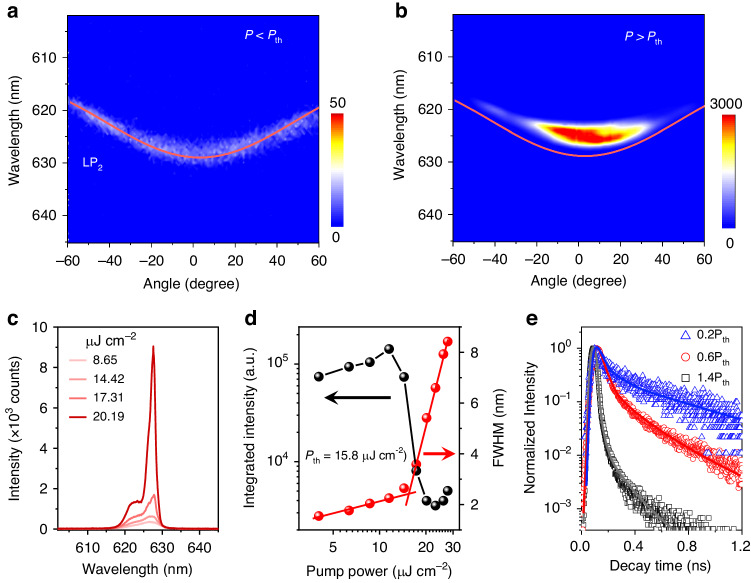
Polariton lasing emission. Angle-resolved PL spectrum measured at *P* < *P*_th_ (**a**) and *P* > *P*_th_ (**b**). **c** PL spectra of LP_2_ branch at the different pump densities. **d** The integrated PL intensities (red dots) and line width (black dots) of the LP_2_ branch as a function of pump density. **e** The PL decay profiles under different pump densities

The behaviors of EP lasing in the above TTPSB OPELD are investigated using a focused second harmonic (*λ* = 400 nm, pulse width 150 fs, 1 kHz) of a Ti:sapphire regenerative amplifier. Figure 2a shows the angle-resolved PL (ARPL) spectrum measured at a low pump density of *P* = 8.65 μJ cm^−2^, which perfectly coincides with the experimental and calculated LP_2_ dispersion (red line). This demonstrates that the ARPL signals originate from the EP emission. In strong contrast to the fact that EPs populate the entire LP_2_ branch at low pump density, they condense at the bottom of the LP_2_ branch within the angle of around *θ* = ±20° when the pump density increases to *P* = 20.19 μJ cm^−2^ (1.2 *P*_th_, Fig. 2b). As shown in Fig. 2c, the PL spectra exhibit typical lasing behaviors, with an explosive increase in intensity and FWHM narrowing above a critical threshold. The clear power-dependent blueshift of the lasing spectra is visible above the *P*_th_ (Fig. 2c). We plot the integrated intensity (red dots) and FWHM (black dots) of PL spectra as a function of pump density in Fig. 2d. The intensity dependence is separately fitted to power laws *x*^*p*^ with *p* = 0.41 ± 0.04, and 6.16 ± 0.19, respectively. The threshold is determined to be *P*_th_ = 15.8 μJ cm^−2^ located at the intersection between the sublinear and superlinear regions. Meanwhile, the FWHM narrows from 8 nm to 2 nm. Figure 2e shows the time-resolved PL from the LP_2_ emission measured with a streak camera. At low pump density *P* = 0.2 *P*_th_ (3.16 μJ cm^−2^), the PL follows single exponential decay with τ = 0.36 ± 0.01 ns. Above the threshold, e.g., *P* = 1.4 *P*_th_ (22.12 μJ cm^−2^), the PL decay time collapses sharply to <30 ps, which is limited by the resolution of our apparatus. This clear EP lasing with a low threshold lays the foundation for evaluating the possibility of realizing electrically pumped EP lasers.

### Polariton EL characterization

**Fig. 3:**
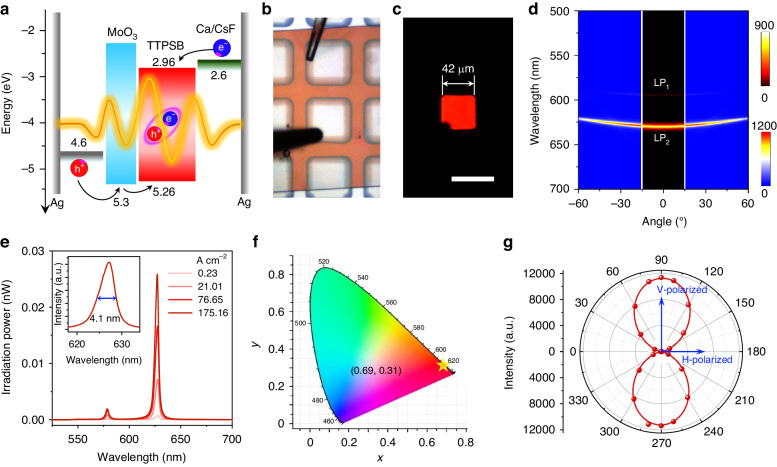
Polariton EL from TTPSB OPLED. **a** Energy diagram for multilayered components in the device. **b** Bright-field and **c** EL images of TTPSB OPLED. Scale bar: 50 μm. **d** ARPL spectrum and AREL spectrum (in the black box). **e** EL spectra at the different current densities. **f** CIE coordinates and **g** polarization angle-dependent EL characteristics of LP_2_ branch

The above TTPSB OPLED is also directly applied to EL emission. Figure 3a presents the energy diagram of this OPLED (also see details in Schemes S2 and S3). Here, MoO_3_ and CsF were used as the hole- and electron-injection materials for efficient carrier injection, which match well with the highest occupied molecular orbital (HOMO) level (−5.26 eV) and the lowest unoccupied molecular orbital (LUMO) level (−2.96 eV) of TTPSB (Fig. S5), respectively. We also fabricated the devices dedicated to pure electron and pure hole transport and subsequently measured their space-charge-limited current (Fig. S6). The calculated electron mobility, *μ*_e_ = 4.72 × 10^−7^ cm^2^ V^−1^ s^−1^ and the hole mobility, *μ*_h_ = 2.42 × 10^−5^ cm^2^ V^−1^ s^−1^, respectively.

We further carried out the EL measurements using a sourcemeter (Keithley 2400) equipped with the detection system in a nitrogen-filled glovebox (details in Supplementary Materials). Figure 3b presents the bright-field optical image of the chosen OPLED. The bright and pure red-color EL is observed from the top electrode (Fig. 3c). The angle-resolved EL (AREL) spectrum is collected and presented in Fig. 3d, comparing with the ARPL of the same OPLEDs. Notably, the angle range of the AREL spectrum is about ±15° limited by the numerical aperture of the microscope lens in our setup. As shown in Fig. 3d, the EL emission in the AREL spectrum agrees well with the dispersions of LP_1_ and LP_2_ in the ARPL spectrum, illustrating that the EL signals stem from the polariton emission. We integrated the AREL spectra within the ±15° range and observed a narrow full width at half maximum (FWHM) of only 4.1 nm (inset of Fig. 3e). Additionally, we collected full-angle emission spectra of the microcavity (Fig. S7) and revealed a FWHM of only 9.1 nm, significantly narrower than mainstream narrowband emitting OLED devices^2,10^. This suggests that no color change is noticeable in our OPLEDs in comparison with the severe angular dispersion in traditional microcavity OLEDs^15^. This EP emission with slight angular dispersion might benefit from the heavy exciton-like polaritons in our H-aggregate microcrystals, which leads to angle-independent narrow EP emission. Figure 3e shows the power-dependent EL spectra of this OPLED. The main peak of EP emission increases in intensity and maintains its FWHM during the process of increasing the pump current density. In our case, this narrow emission provides a high color purity as indicated by the CIE coordinates of (0.69, 0.31), as shown in Fig. 3f, which is quite close to the BT.2020 red-light standard (0.708, 0.292).

The highly polarized EP and cavity modes from TTPSB-based OPLEDs indicate its great potential for highly polarized EP EL emission. We perform the polarization angle-resolved EL measurements by inserting an angle-changeable polarizer. The EL intensity at 627 nm (LP_2_ branch) of OPLEDs strongly depends on the polarization angles, as evidenced by the polar-coordinate diagram (Figs. 3g and S8) from the polarized EL spectra of representative polarization angles. The EL intensity of the OPLED has a maximum value along V-polarization and a minimum value along H-polarization. The polarization ratio is calculated to be as high as 170 from PR = *I*_V_/*I*_H_^33^, where *I*_V_ and I_H_ are the intensities of V- and H-polarized EL at the LP_2_ branch, respectively.

**Fig. 4:**
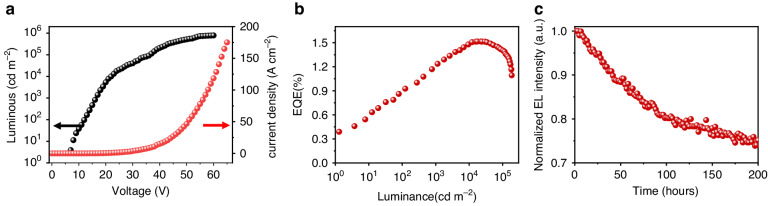
TTPSB OPLED performances. **a** Current density and luminance versus voltage. **b** EQE versus luminance and **c** EL intensity decay as a function of operational time at an initial luminance of 1000 cd m^−2^

Figure 4 summarizes the characteristics of this optimized TTPSB OPLED (the optimization process is detailed in Table S3). The maximum luminance and current efficiency of about 780,000 cd m^−2^ and a maximum current density of about 175 A cm^−2^, respectively, were achieved (Fig. 4a), which is greatly superior to the other OPLEDs reported so far^15^. According to the ARPL and AREL of this TTPSB OPLED, the optimized EQE is determined to be a maximum of 1.52% at 23,000 cd m^−2^ (Fig. 4b). We estimated the theoretical efficiency of our TTPSB OLEDs according to the equation of EQE = *χ* × *γ* × Φ_S_ × *η*_out_, which is widely adopted in OLED, where the *χ* fraction of spin statistics is 25% for fluorescence materials in the EL process, *γ* is the charge balance or the exciton formation efficiency (ideally 100%), Φ_S_ for TTPSB is 17.7%, and the *η*_out_ light out-coupling factor is usually 20% for thin-film devices. Thus, the ideal efficiency of TTPSB OLEDs without microcavity is estimated to be 0.88%. Here, the experimental measured EQE of 1.52% is higher than the calculated value of 0.88%, mainly attributed to the fact that the output coupling efficiency in our case exceeds the 20% upper limit observed in thin film samples because the horizontal orientation of TTPSB molecules in the cavity affects the light outcoupling. Figure 3b displays our TTPSB OPLEDs have a low-efficiency roll-off and high stability, which might be due to the efficient avoidance of exciton quenching at high current density by short-lived polaritons. The OPLED lifetime is characterized by an initial luminance of 1000 cd m^−2^ and plotted as a function of time in Fig. 4c. An outstanding 95% of the initial luminance (LT_95_), the key indicator for verifying device stability, is 19.9 h. Moreover, after 200 h of operation, the luminance is still maintained at more than 70% of the initial luminance.

## Discussion

Regrettably, we do not observe electrically pumped polariton lasers in our OPLEDs. However, the fact of the simultaneous realization of an optically pumped laser and polariton EL allows us to estimate the required polariton density for an electrically pumped laser. For the case of optical pumping, the density of pumped exciton (*N*_th_) is deduced from the equation *N*_th_ = 0.5 *P*_th_/[*z*(*hc*/*λ*)]^34^, where 0.5 is the transmittance of the pump laser to the top reflector, *P*_th_ is the threshold of the polariton laser, z is the thickness of the TTPAB microcrystal, *h* is the Planck constant, *c* is the velocity of light in a vacuum, and *λ* is excitation wavelength. The optically pumped rate (*P*_OP_) of the reservoir is estimated by the equation *P*_OP_ = *N*_th_/*T*_PW_, where *T*_PW_ is the pulse width of the pump laser, *P*_OP_ ~ 4 × 10^26^ cm^−3^ s^−1^. The rate equation approach is used to estimate the generating polariton density in the LP branch. First, the exciton density in the reservoir *n*_R_ is depicted by

1 $${\frac{{d}_{{n}_{{\rm{R}}}}}{{{\rm{d}}t}}={P}_{{\rm{OP}}}-\frac{{n}_{{\rm{R}}}}{{\tau }_{{\rm{therm}}}}-\frac{{n}_{{\rm{R}}}}{{\tau }_{{\rm{nr}}}}}$$

where *τ*_therm_ is the upper limit of relaxation time from the reservoir into the LP branch, which is approximately equal to the polariton lifetime (~40 fs, estimated by the FWHM of LP branches). The non-radiative exciton lifetime *τ*_nr_ is calculated to be 0.52 ns. The polariton density *n*_LP_ in the LP branch is given by

2 $${\frac{{d}_{{n}_{{\rm{LP}}}}}{{{\rm{d}}t}}=\frac{{n}_{{\rm{R}}}}{{\tau }_{{\rm{therm}}}}-\alpha \frac{{n}_{{\rm{LP}}}}{{\tau }_{{\rm{cav}}}}-\beta \frac{{n}_{{\rm{LP}}}}{{\tau }_{{\rm{nr}}}}}$$

where *τ*_cav_ is the photon lifetime (~19 fs, estimated by the FWHM of the uncoupled cavity modes). *α* and *β* are the photonic fraction and excitonic fraction of polariton, respectively. Assuming quasi-steady-state conditions $({{\rm{d}}n}/{{\rm{d}}t}\approx 0)$, the critical polariton density is calculated as^28^

3 $${{n}_{{\rm{LP}}}=\frac{{P}_{{\rm{OP}}}}{{\tau }_{{\rm{therm}}}}{\left(\frac{\alpha }{{\tau }_{{\rm{cav}}}}+\frac{\beta }{{\tau }_{{\rm{nr}}}}\right)}^{-1}{\left(\frac{1}{{\tau }_{{\rm{therm}}}}+\frac{1}{{\tau }_{{\rm{nr}}}}\right)}^{-1}}$$

to be ~2 × 10^13^ cm^−3^ for optically pumped polariton laser, which is of the same order of magnitude as anthracene^35^.

In the case of electrical pumping, the pumped rate is determined (*P*_EP_) to be ~6 × 10^26^ cm^−3^ s^−1^ at the highest current density of our devices. The *χ* fraction of spin statistics, which is 25% for fluorescence materials, is introduced to modify the *P*_EP_. The electrically pumped polariton density in the LP is defined as

4 $${n}_{{\rm{LP}}}=\frac{\chi {P}_{{\rm{EP}}}}{{\tau }_{{\rm{therm}}}}{\left(\frac{\alpha }{{\tau }_{{\rm{cav}}}}+\frac{\beta }{{\tau }_{{\rm{nr}}}}\right)}^{-1}{\left(\frac{1}{{\tau }_{{\rm{therm}}}}+\frac{1}{{\tau }_{{\rm{nr}}}}\right)}^{-1}$$

The required *n*_LP_ for electrically pumped lasing is calculated to be 5 × 10^12^ cm^−3^, which is four times lower than that of optical pumping. Therefore, in order to achieve the electrically pumped polariton laser, we must improve the *n*_LP_. We think that the enhancement of the OPLED can be approached from two aspects. Firstly, the highly reflective distributed Bragg reflector (DBR) mirrors should replace the metal mirrors to increase the quality factor to over 1000, as this has been shown to improve the *τ*_therm_ and reduce the threshold. Secondly, the *χ* fraction could be increased to 100% through the utilization of thermally activated delayed fluorescence (TADF) or phosphorescent materials that can efficiently utilize triplet excitons.

## Conclusion

In summary, we demonstrate a high-luminance, narrow-emission, and high-polarized OPLED that presents excellent optically pumped polariton laser characteristics. The device exhibits a pure-red-color emission with a high luminance of over 780,000 cd m^−2^, high color purity of (0.69, 0.31), a highly polarized degree of 170, and remarkable long-term stability. We further evaluate the potential for electrically pumped polariton lasers through theoretical analysis and provide possible solutions. This work provides a powerful strategy with a material–device combination that paves the way for electrically-driven OSC-based polariton luminescent devices and possibly lasers.

## Materials and methods

### The preparation of TTPSB microcrystals

In our experiment, 1,4-dimethoxy-2,5-di(2,2’,5’,2”-terthiophenestyryl) benzene (TTPSB) microbelts were fabricated using a facile physical vapor deposition (PVD) method. A quartz boat carrying 3 mg TTPSB was then placed in the center of a quartz tube which was inserted into a horizontal tube furnace. A continuous flow of cooling water inside the cover caps was used to achieve a temperature gradient over the entire length of the tube. To prevent oxidation of TTPSB, argon (Ar) was used as an inert gas during the PVD process (flowrate: 15 sccm min^−1^). The pre-prepared hydrophobic substrates were placed on the downstream side of the argon flow for product collection, and the furnace was heated to the sublimation temperature of TTPSB (at a temperature region of ~320 °C), upon which it was physically deposited onto the pre-prepared hydrophobic substrates at temperature region of ~230 °C for 1 h.

### The preparation of TTPSB-crystal-based microcavity

Highly n-doped (100) Si wafers (0.05–0.2 U cm) were successively cleaned with piranha solution (70/30 vol./vol. H_2_SO_4_/H_2_O_2_), deionized water, and isopropanol, respectively, and then were dried by N_2_ and O_2_ plasma for 10 min. The bottom electrode was fabricated by using a metal vacuum deposition system (Amostrom Engineering 03493) to thermally evaporate a silver film with a thickness of 150 ± 5 nm (reflectivity *R* ≥ 99%) on the glass substrate, the root mean square roughness (*R*_q_) of the silver film in the 5 μm × 5 μm area is 2.23 nm, a 5 ± 0.4 nm MoO_3_ layer was deposited on the silver film with *R*_q_ of 0.4 nm, the deposited rates were both 0.2 Å s^−1^ and the base vacuum pressure is 3 × 10^−6^ Torr. The TTPSB crystals were transferred to the substrate for the next step of device preparation. We used a shadow mask to cover the crystals for evaporating top electrodes. Finally, cesium fluoride (CsF) with 2 ± 0.2 nm thickness was used as the modification layer between crystals and calcium (thickness of 10 ± 1 nm) to improve the electron injection, and then a 30 ± 2 nm Ag layer was deposited as top reflector.

### Structural and spectroscopic characterization

As-prepared TTPSB microbelts were characterized by field emission scanning electron microscopy (FE-SEM, HITACHI S-4800) by dropping them on a silicon wafer. The X-ray diffraction (XRD, Japan Rigaku D/max-2500 rotation anode X-ray diffractometer, graphite monochromatized CuK_α_ radiation (*λ* = 1.5418 Å)) operated in the 2*θ* range from 3° to 30°, by using the samples on a cleaned glass slide.

The fluorescence micrograph, diffused reflection absorption, and emission spectra were measured on Olympus IX71, HITACHI U-3900H, and HITACHI F-4600 spectrophotometers, respectively. The photoluminescence spectra of isolated single TTPSB microbelts in microcavity were characterized by using a homemade photoelectric integrated detection system equipped with a ×50 0.42 NA objective lens (working distance 20.5 mm) (Scheme S1). The second harmonic (*λ* = 400 nm, pulse width 150 femtosecond) of a 1 kHz Ti: sapphire regenerative amplifier was focused to a 50-μm diameter spot to excite the selected single TTPSB on a two-dimensional (2D) movable table. A sourcemeter (Keithley 2400) equipped with probes is applied to electrical measurement.

### Calculation of EQE and irradiation power

The electrically driving light emission from OLEDs was detected by tailor-made equipment for recording emission image, spectra and current simultaneously.

Formulaically speaking, according to the following definition Eq. (1), the EQE of EL device is acquired from the number of the collection emissive photons *n*_ν_ divided by the number of injected carriers *n*_e_

5 $${\rm{EQE}}=\frac{{n}_{{\rm{v}}}}{{n}_{{\rm{e}}}}$$

A halogen lamp with a known irradiation power of *P*c(*λ*), which is calibrated by the Shanghai Institute of Measurement and Testing Technology, is used as the calibration light source. A reflector with a known reflectivity of *R*(*λ*) is placed at the focal plane of the lens, and the light intensity by a CCD camera (PIXIS 100 BR) equipped spectrometer after being reflected by the reflector is *I*_c_(*λ*), the response function of the light path to light of different wavelengths is *H*(*λ*) = *I*_c_(*λ*)/*P*_c_(*λ*) × *R*(*λ*). When testing electroluminescence samples, the obtained irradiation power at a specific wavelength is *P*_s_(*λ*) = *I*_s_(*λ*)/*H*(*λ*), where *I*_s_(*λ*) is the electroluminescence intensity measured by the same detection light path and the spectrometer.

The distribution of photon number *n*_ν_, is calculated by Eq. (2). Hence, the number of total photons *n*_ν_ from detected emissive spectra ranging from starting and ending wavelength can be calculated through mathematical integral.

6 $${n}_{{{v}}}=\int \frac{{P}_{{\rm{S}}}\left(\lambda \right)\lambda {\rm{d}}\lambda }{{hc}}$$

where *n*_ν_ is the number of photons at any wavelength, in counts. *λ* is the wavelength of the emissive light, in nm. *h* is Planck constant (6.626 × 10^−34^ J s). *c* is the velocity of light in a vacuum (3.0 × 10^8^ m s^−1^). The number of injected carriers ne during the test is calculated from the recombination current *I* at saturation region of OLED devices divided by the elementary charge *e* = 1.602 × 10^−19^ C and integral time.

Therefore, the EQE is calculated by

7 $${\rm{EQE}}=\frac{\int {P}_{{\rm{S}}}\left(\lambda \right)\times \frac{\lambda }{{hc}}{\rm{d}}\lambda }{A/e}$$

### Supplementary information

Supporting Information Organic Polaritonic Light-Emitting Diodes with High Luminance and Color Purity toward Laser Displays
