# Supplementary material for: Organic polaritonic light-emitting diodes with high luminance and color purity toward laser displays
Source: Light Sci Appl. 2024 Aug 15;13:191. doi: 10.1038/s41377-024-01531-0 (PMC11327354; doi:10.1038/s41377-024-01531-0)
Supplement: Supplementary file 1 — Supporting Information Organic Polaritonic Light-Emitting Diodes with High Luminance and Color Purity toward Laser Displays [file 41377_2024_1531_MOESM1_ESM.docx]

**Supporting Information**

**Organic Polaritonic Light-Emitting Diodes with High Luminance and Color Purity toward Laser Displays**

Jianbo De,^1,2,†^ Ruiyang Zhao,^1,†^ Fan Yin,^1,†^ Chunling Gu,^3^ Teng Long,^1^ Han Huang,^4^ Xue Cao,^1^ Cunbin An,^1^ Bo Liao,^5^ Hongbing Fu,^1,5,^* Qing Liao,^1,^*

^1^Beijing Key Laboratory for Optical Materials and Photonic Devices, Department of Chemistry, Capital Normal University, Beijing 100048, China

^2^Beijing Special Engineering Design and Research Institute, Beijing 100028, China

^3^Institute of Process Engineering, Chinese Academy of Sciences, Beijing, 100190, China

^4^Institute of Molecule Plus, Tianjin University, and Collaborative Innovation Center of Chemical Science and Engineering (Tianjin), Tianjin 300072, China

^5^School of Materials Science and Engineering, Hunan University of Science and Technology, Xiangtan, Hunan 411201, China

^†^These authors contributed equally to this work.

*Corresponding author. Email: hbfu@cnu.edu.cn; liaoqing@cnu.edu.cn


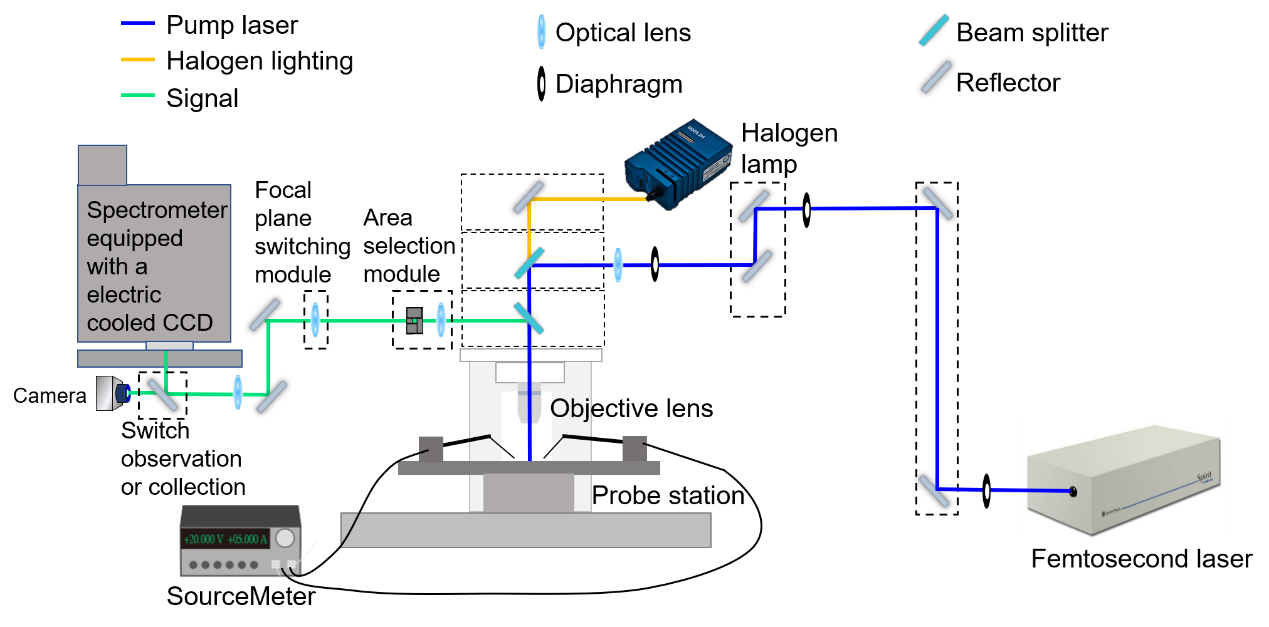


**Scheme. S1.** Schematic demonstration of the experimental setup for the photoelectric characterization. In order to maintain stability, the whole set of equipment is placed on the optical platform. The blue line represents the pump laser, the yellow line represents the white light path origin from a halogen lamp and the green line is the collection and observation path.

**
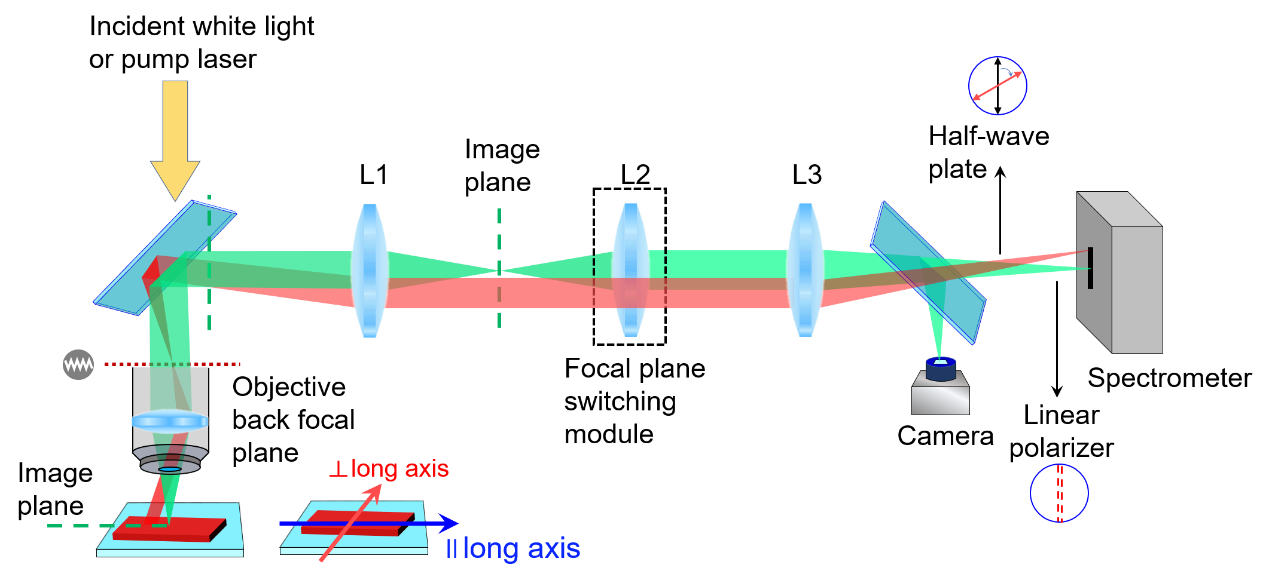
**

**Scheme. S2.** Experimental setup allowing to obtain polarization-resolved spectroscopy. L1-L3: lenses. The red beam traces the optical path of the reflected light from the sample at a given angle. The green beam traces the optical path of the image plane.

The angle-resolved spectroscopy was performed at room temperature by the Fourier imaging using a long working distance lens, 50 × objective lens of a NA 0.42, corresponding to a range of collection angle of ± 15°, for electrical characterization. A short working distance lens, 100 × objective lens of a NA 0.95 is used to recording the ARPL and Angle-resolved reflectivity (ARR) with a broader angle of ± 60°. An incident white light from a Halogen lamp with the wavelength range of 400 - 700 nm was focused on the area of the microcavity containing a TTPSB microcrystal. The k-space or angular distribution of the reflected light (red light path in Scheme S2) was located at the back focal plane of the objective lens. The L2 is a focal plane switching lens, used for switching the right focal plane of L3 between image plane and back focal plane. When the L2 is out, Lenses L1 and L3 formed a confocal imaging system together with the objective lens, by which the *k*-space light distribution was imaged at the right focal plane of L3 on the entrance slit of a spectrometer equipped with an electric-cooled CCD. When the L2 is inserted, the right focal plane of L3 is converted to image plane which can be recorded by camera.

In order to investigate the polarization properties, we placed a linear polarizer, a half-wave plate in front of spectrometer to obtain the polarization state in the horizontal-vertical (0° and 90°), horizontal-polarization (0°) is parallel to long axis of the TTPSB crystal, vertical-polarization (90°) is perpendicular to long axis of the TTPSB crystal.

**Polariton dispersion**

The polariton dispersion in Fig. 1b was calculated by a coupled harmonic oscillator Hamiltonian (CHO) model (S. Kena-Cohen *et al.*, *Phys. Rev. Lett.* 2008, 101, 116401.). The 2×2 matrix in equation (1) below describes the CHO Hamiltonian:

$\left( \begin{matrix} E_{CMn}(\theta) & \Omega/2 \\ /2 & E_{X} \end{matrix} \right)\binom{\alpha}{\beta}=E\binom{\alpha}{\beta}$ (1)

Where *θ* represents the polar angle, *E*_CMn_(*θ*) is the cavity photon energy of the *n*^th^ cavity mode as a function of *θ*, *E*_X_ is the exciton 0-0 absorption energy of DPAVBi microbelts at 2.67 eV (464 nm) and Ω (eV) denotes the coupling. The magnitudes |α|^2^ and |β|^2^ correspond to the photonic and the excitonic fraction, respectively.

The cavity photon dispersion is given by:

$E_{\mathrm{CMn}}\left( \theta\right)=\sqrt{\left( E_{c}^{2}\times\left( 1-\frac{\sin^{2} \theta}{n_{eff}^{2}} \right)^{-1} \right)}-\left( n-1 \right)\times l$ (2)

where $E_{c}$ represents the cavity modes energy at *θ* = 0°, $E_{CM1}\left( \theta\right)$ represents the energy of the first cavity mode when n = 1, (n-1) × *l* represents the energy difference from the first cavity mode. The theoretical fitting dispersion of the uncoupled H-polarized cavity modes (*n*_eff_ = 1.60, cyan dotted lines), uncoupled V-polarized cavity modes (*n*_eff_ = 2.10, green dotted lines) and coupled lower polariton branches (red dotted lines) are shown in Fig. S3. Diagonalization of this Hamiltonian yields the eigenvalues, *E*_±_(*θ*), which represents the upper and lower polariton (UP and LP) in-plane dispersions (H. Deng *et al.*, *Rev. Mod. Phys.* 2010, **82**, 1489-1537.),

$E_{\pm}\left( \right)=\frac{E_{X}+E_{\mathrm{CMn}}()}{2}$±$\frac{1}{2}$ $\sqrt{\left( E_{X}-E_{\mathrm{CMn}}\left( \right) \right)^{2}+\hbar^{2}\Omega^{2}}$ (3)

**Crystal characterization**

**
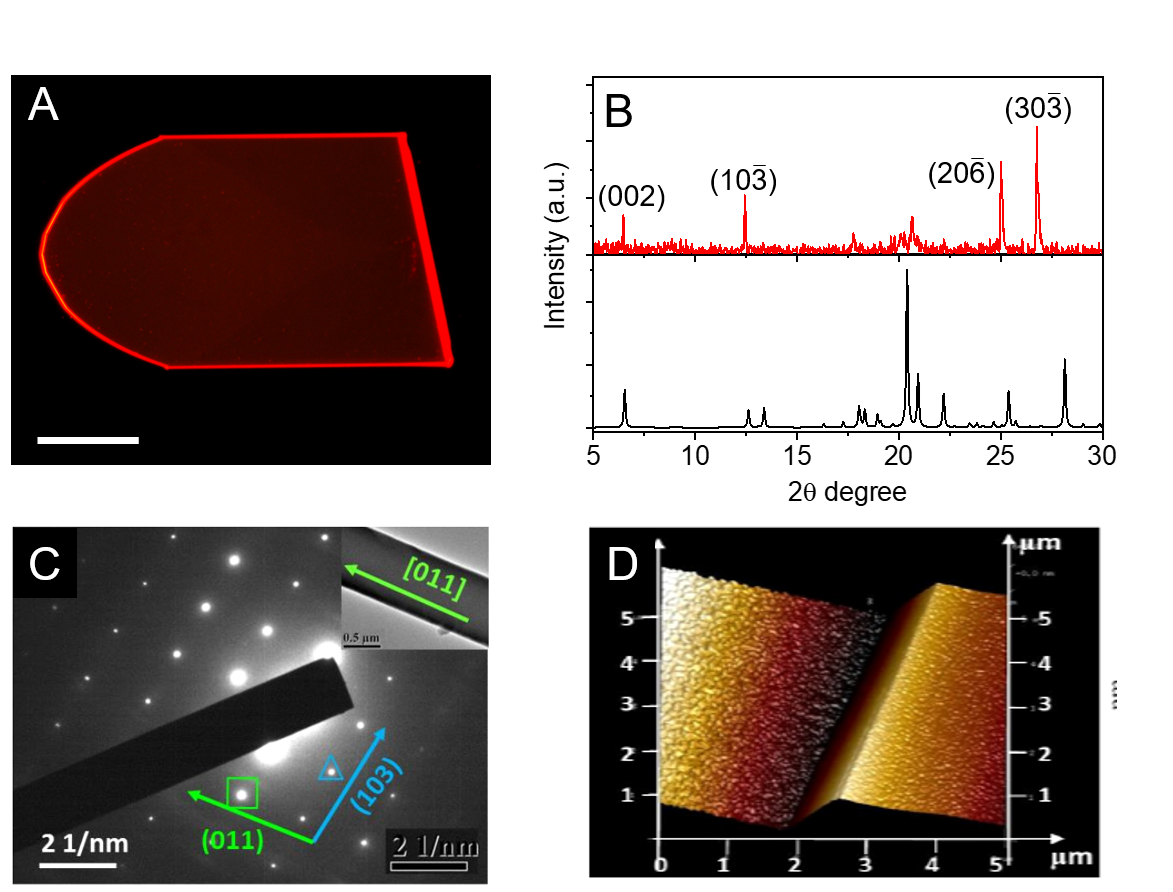
**

**Figure. S1.** (a) Fluorescence microscopy image of as-prepared TTPSB microcrystal, which shows a typical length of about 200 μm and width of 150 μm. (b) X-ray diffraction (XRD) spectrum of TTPSB microbelts and powder. (c) Selected area electron diffraction (SAED) pattern recorded by directing the electron beam perpendicular to the top-surface of a single TTPSB microcrystal. Inset: the corresponding transmission electron microscopy (TEM) image. These reveal that the TTPSB microbelt is single crystalline. (D) Atomic force microscopy (AFM) image of as-prepared TTPSB microbelts.

PL image and AFM image depict that typical large-scale TTPSB microbelts with smooth surface and sharp edge have been successfully fabricated (Figure S1a). Their length (*l*) is found to range between 170 and 230 μm, while the width (*w*) and the height (*h*) is determined to be about 120 μm and 580 nm, respectively, according to AFM measurements. XRD spectrum is dominated by a series of diffraction peaks corresponding to crystal plane (10$\bar{\text{3}}\text{)}$ with *d* = 7.011 Å, indicating that TTPSB microcrystals are bordered by (10$\bar{\text{3}})$ and ($\bar{\text{1}}\text{03)}$ crystal planes on the top and bottom surfaces. The observed sharp spots in selected area electron diffraction (SAED) pattern (Figure S1C) clarifies that these microbelts are single crystalline in nature. According to that monoclinic TTPSB crystals belong to the space group of P2_1_/n, with cell parameters of *a* = 10.4006 Å, *b* = 5.5454 Å, *c* = 27.0574 Å, *α* = *γ* = 90°, and *β* = 93.409° (CCDC No. 1554859, Table S1), the blue triangle spot in Figure S1C is ascribed to (103) Bragg reflections with a *d*-spacing value of 13.558 Å, and the red square spot corresponds to (011) crystal plane with *d*-spacing value of 11.328 Å, in good agreement with the cell parameters of this monoclinic crystal structure.

**Table. S1.** Crystal data and structure refinement for TTPSB.

| Sample | TTPSB |
| --- | --- |
| Space Group | P 2_1_/n |
| *a* | 10.4006 Å |
| *b* | 5.54540 Å |
| *c* | 27.0574 Å |
| *α* | 90.000º |
| *β* | 93.409º |
| *γ* | 90.000º |


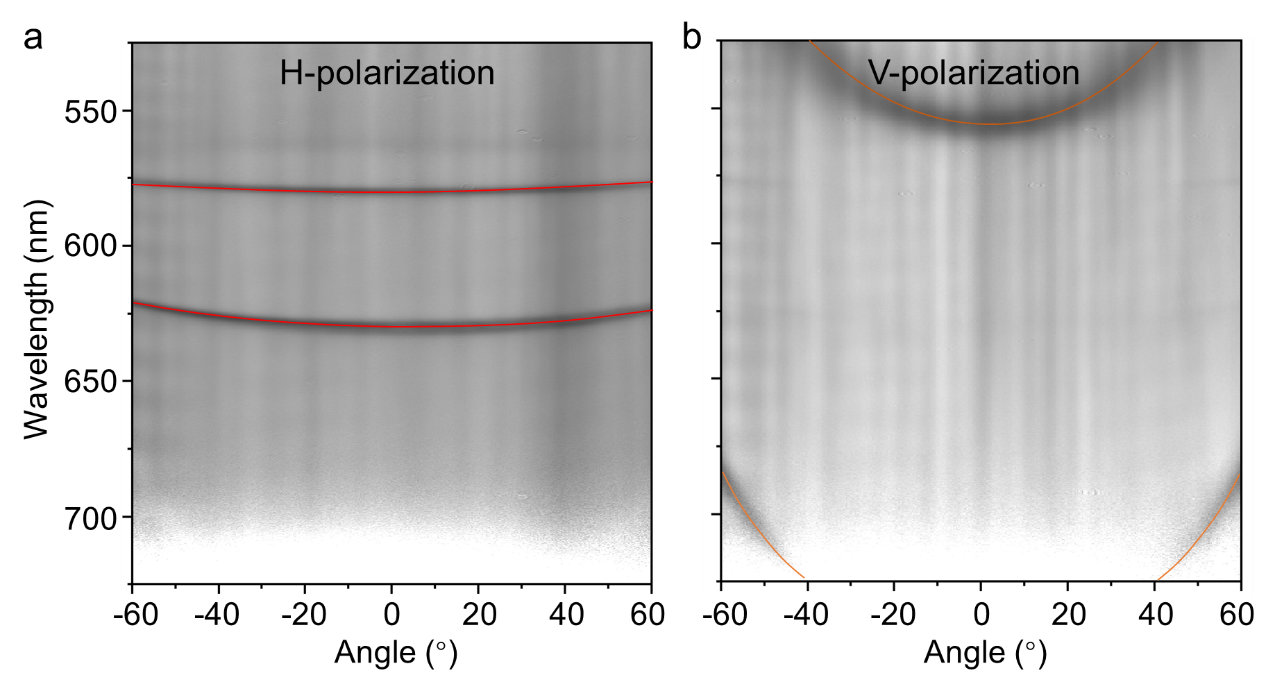


**Figure. S2.** Measured *k*-space angle-resolved reflectivity spectra of a selected microcavity at room temperature in horizontal (H) polarization (a) and vertical (V) polarization (b) along X-direction of the single-crystal cavity.

**Table. S2.** Coupled Harmonic Oscillator Model Fitting Results for OPLEDs.

| Coupling mode | LP_1_ | LP_2_ |
| --- | --- | --- |
| Rabi splitting (mev) | 450 | |
| Detuning (mev) | 568 | 57 |
| \|α\|^2^ | 0.04 | 0.38 |
| \|β\|^2^ | 0.96 | 0.62 |
| exciton | 2.164 | 2.119 |


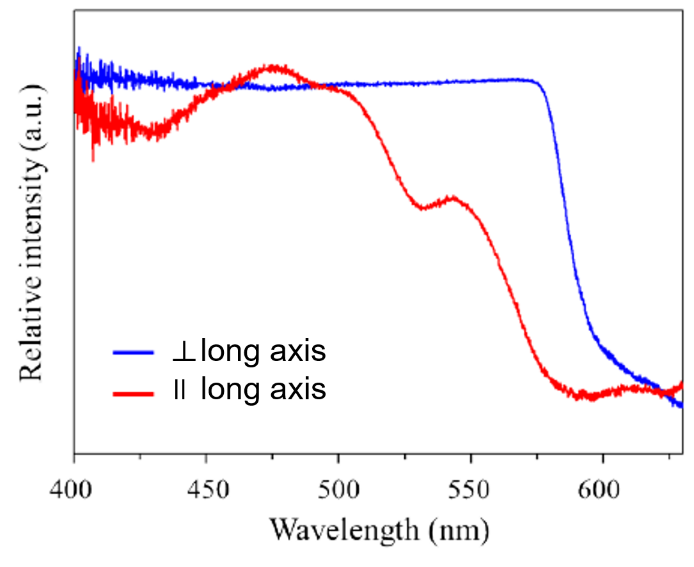


**Figure. S3.** Polarization-dependent absorption spectra of a single TTPSB microbelt. A broad and intense absorption peak is observed (blue line) when the polarization of the white light from a halogen lamp is adjusted to be perpendicular to the long axis of the microbelt, whereas the absorption is much weaker (red line) when the polarization of the white light is parallel to the long axis of the microbelt. The descriptions of polarization directions also see Scheme S2. These distinct polarization-dependent absorption features are consistent with the fact that the anisotropy is a result of the highly ordered uniaxial alignment of TTPSB molecules in single crystalline microbelts.


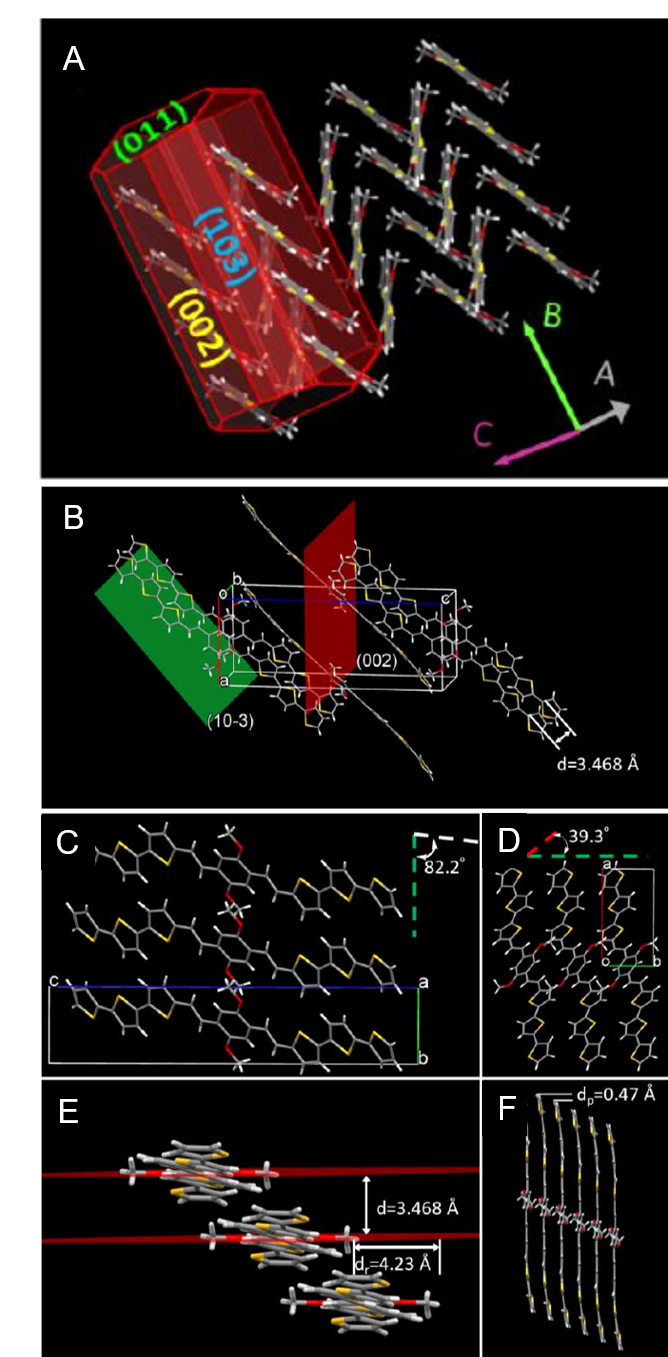


**Figure. S4.** (A) Simulated growth morphology of TTPSB molecules using Material Studio package. (B) Molecular packing arrangement of TTPSB in the nanowires, viewed almost along the crystal *b*-axis. (C) Viewed normal to *bc* plane with illustration of pitch angle. (D) Viewed normal to *ab* plane with illustration of roll angle. (E) Viewed the shortest separation of *d* = 3.468 Å and the transverse displacement of *d*_r_ = 4.23 Å. (F) Viewed the longitudinal displacement of *d*_p_ = 0.47 Å.

Combining the analysis of SAED, TEM and XRD results, TTPSB molecules within 1D microbelts adopt a herringbone packing arrangement and stack co-facially along the crystal [011] plane with the shortest separation about 3.47 Å, which indicates a typical π-π stacking (Figure.S4c). Further analyze the molecules arrangement (Figure.S4d-g), the pitch angle (which defines the angle between the molecular transition dipole and the π-stack direction) of 82.2° (corresponding to the longitudinal displacements between neighboring molecules of 0.47 Å) ensures co-facially π-stacking, which might be beneficial to efficient charge transport channel for 1D microbelts. While the roll angle of 39.3° (corresponding to the transverse displacements 4.23 Å) greatly reduces the quenching effect caused by the π-π interaction and brings strong PL emission. According to the molecular exciton model (F. Würthner et. al, *Angew. Chem. Int. Ed.* 2011, 50, 3376-3410.), the pitch angle is < 54.7° in a J-type aggregate and > 54.7° in an H-type aggregate. Therefore, it is expected that H-type coupling occurs in TTPSB microbelts.

**
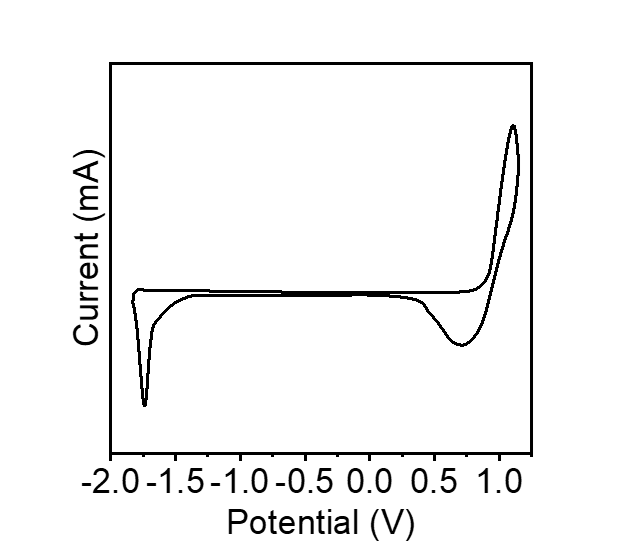
**

**Figure. S5.** Cyclic voltammetry (CV) curve of DBTVB, the inset is: CV measurement of ferrocene as reference. *E*_HOMO_ = -(*E*_oxonset_ + 4.8 - *E*_reFc/Fc+_) = -(1.2 + 4.8 - 0.7385) = -5.26 eV, *E*_LUMO_= *E*_HOMO_ + *ΔE*_g_ =-5.26 eV + 2. 3 eV = - 2.96 eV

**
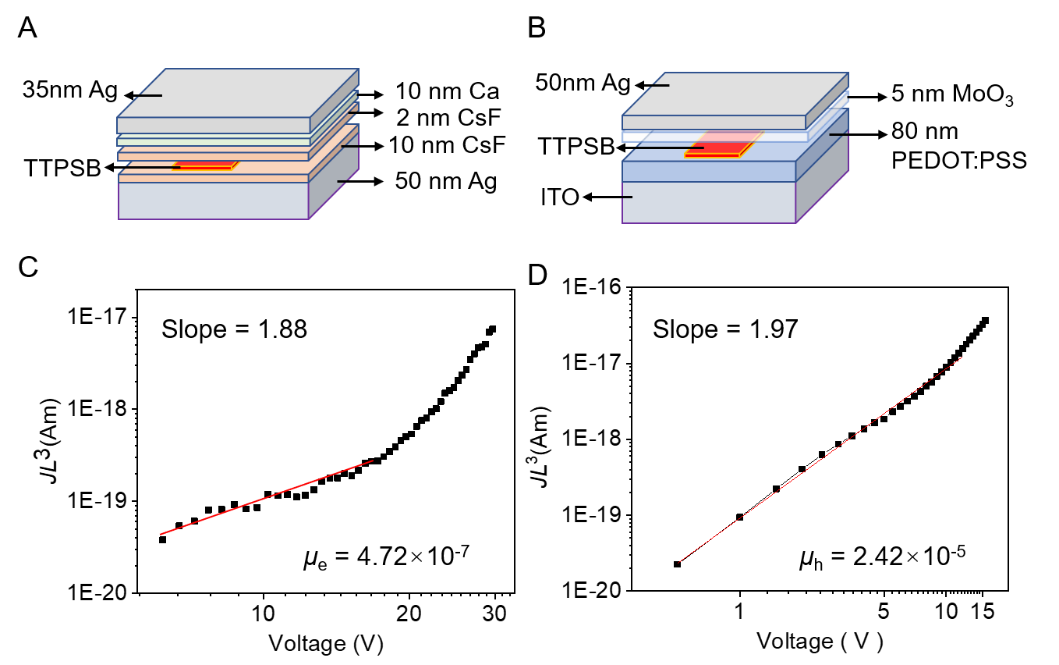
**

**Figure. S6.** A and B present the structural schematics of the pure electron and pure hole devices, respectively. C and D depict the relationship between the current density (*JL*^3^) and voltage (V) for the pure electron and pure hole devices, respectively.

**
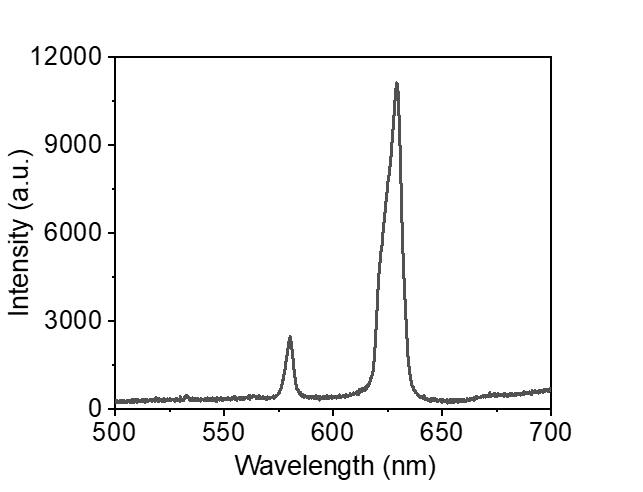
**

**Figure. S7.** The non-angle-resolved emission spectrum of the TTPSB microcavity.


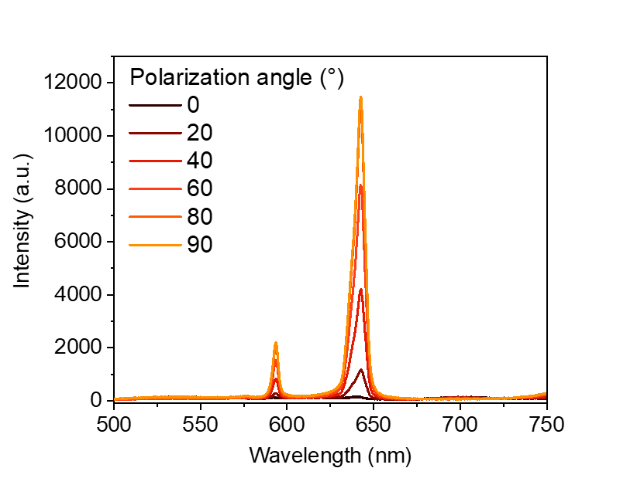


**Figure. S8.** The polarized spectrum of 0° to 90°. The spectrum at 0° is corresponding to the H-polarized, 90° is V-polarized.

**Table. S3**. The impact of the injection layer thickness on device performance

| Injection layer | Turn-on voltage | Luminous _max_ | EQE _max_ |
| --- | --- | --- | --- |
| 7 nm MoO_3_ 4 nm CsF | 8 V | 268267 cd m^-2^ | 0.71 % |
| 5 nm MoO_3_ 4 nm CsF | 9 V | 338946 cd m^-2^ | 0.88 % |
| 2 nm MoO_3_ 4 nm CsF | 7 V | 109240 cd m^-2^ | 0.26 % |
| 5 nm MoO_3_ 3 nm CsF | 13 V | 453094 cd m^-2^ | 0.86 % |
| 5 nm MoO_3_ 2 nm CsF | 9 V | 783094 cd m^-2^ | 1.52 % |
| 5 nm MoO_3_ 1 nm CsF | 10 V | 644998 cd m^-2^ | 0.75 % |
